# Supplementary material for: The Dietary Intake and Practices of Adolescent Girls in Low- and Middle-Income Countries: A Systematic Review
Source: Nutrients. 2018 Dec 14;10(12):1978. doi: 10.3390/nu10121978 (PMC6315365; doi:10.3390/nu10121978)
Supplement: Supplementary file 1 [file nutrients-10-01978-s001.pdf]

The Dietary Intake and Practices of Adolescent Girls in Low and Middle Income Countries: A Systematic Review

Supplementary Materials

Material S1. Search Strategy.

1. *Adolescents*: Exp adolescent/ or exp minors/ or exp pregnancy in adolescence/ or (teen* or youth* or "pre-teen*" or "child-bearing age" or pregnan* or preconception or prenatal or postnatal or "young adult*").mp
2. *Diet/Foods:* Exp diet/ or exp diet, food, and nutrition/ or exp diet surveys/ or exp diet, western/ or exp energy intake/ or exp eating/ or exp food (bread, candy, chocolate, dairy products, butter, ghee, cheese, yogurt, ice cream, margarine, eggs, flour, fruit, meat, poultry, red meat, fish products, nuts, seeds, vegetables)/ or exp beverages (alcoholic, carbonated, coffee milk, tea, water)/ or exp dietary carbohydrates/sucrose/fats/fibre/proteins/ or exp fast foods/ or exp functional food/ or exp food quality/ or exp nutritive value/ or exp nutrition assessment/ or exp nutrition surveys/ or exp portion size/ or exp serving size/ or (“food intake” or “dietary intake” or “nutrient intake” or “food consumption” or “diet diversity” or “diet quality” or “processed foods”).mp
3. *Patterns & Practices:* Exp eating/ or exp drinking/ or exp consumer behaviour/ or exp choice behaviour/ or exp feeding behaviour/ or ex food preferences/ or exp healthy lifestyle or exp meals (breakfast, lunch, dinner, snacks)/ or (“dietary pattern” or “meal pattern” or “eating habit” or “eating custom” or “eating practice” or “nutrition transition”).mp
4. *LMIC:* Developing Countries/ or developing countr*.tw, kf or ("developing country" or "developing countries" or "developing nation" or "developing nations" or "developing population" or "developing populations" or "developing world" or "less developed country" or "less developed countries" or "less developed nation" or "less developed nations" or "less developed population" or "less developed populations" or "less developed world" or "lesser developed country" or "lesser developed countries" or "lesser developed nation" or "lesser developed nations" or "lesser developed population" or "lesser developed populations" or "lesser developed world" or "under developed country" or "under developed countries" or "under developed nation" or "under developed nations" or "under developed population" or "under developed populations" or "under developed world" or "underdeveloped country" or "underdeveloped countries" or "underdeveloped nation" or "underdeveloped nations" or "underdeveloped population" or "underdeveloped populations" or "underdeveloped world" or "middle income country" or "middle income countries" or "middle income nation" or "middle income nations" or "middle income population" or "middle income populations" or "low income country" or "low income countries" or "low income nation" or "low income nations" or "low income population" or "low income populations" or "lower income country" or "lower income countries" or "lower income nation" or "lower income nations" or "lower income population" or "lower income populations" or "underserved country" or "underserved countries" or "underserved nation" or "underserved nations" or "underserved population" or "underserved populations" or "underserved world" or "under served country" or "under served countries" or "under served nation" or "under served nations" or "under served population" or "under served populations" or "under served world" or "deprived country" or "deprived countries" or "deprived

nation" or "deprived nations" or "deprived population" or "deprived populations" or "deprived world" or "poor country" or "poor countries" or "poor nation" or "poor nations" or "poor population" or "poor populations" or "poor world" or "poorer country" or "poorer countries" or "poorer nation" or "poorer nations" or "poorer population" or "poorer populations" or "poorer world" or "developing economy" or "developing economies" or "less developed economy" or "less developed economies" or "lesser developed economy" or "lesser developed economies" or "under developed economy" or "under developed economies" or "underdeveloped economy" or "underdeveloped economies" or "middle income economy" or "middle income economies" or "low income economy" or "low income economies" or "lower income economy" or "lower income economies" or "low gdp" or "low gnp" or "low gross domestic" or "low gross national" or "lower gdp" or "lower gnp" or "lower gross domestic" or "lower gross national" or lmic or lmics or "third world" or "lami country" or "lami countries" or "transitional country" or "transitional countries" or Africa or Asia or Caribbean Region or West Indies or South America or Latin America or Central America or Afghanistan or Albania or Algeria or Angola or Argentina or Armenia or Armenian or Azerbaijan or Bangladesh or Benin or Byelarus or Byelorussian or Belarus or Belorussian or Belorussia or Belize or Bhutan or Bolivia or Bosnia or Herzegovina or Hercegovina or Botswana or Brazil or Bulgaria or Burkina Faso or Burkina Fasso or Upper Volta or Burundi or Urundi or Cambodia or Khmer Republic or Kampuchea or Cameroon or CameroonsOR Cameron or Camerons or Cape Verde or Central African Republic or Chad or China or Colombia or Comoros or Comoro Islands or Comores or Mayotte or Congo or Zaire or Costa Rica or Cote d'Ivoire or Ivory Coast or Cuba or Djibouti or French Somaliland or Dominica or Dominican Republic or East Timor or East Timur or Timor Leste or Ecuador or Egypt or United Arab Republic or El Salvador or Eritrea or Ethiopia or Fiji or Gabon or Gabonese Republic or Gambia or Gaza or Georgia Republic or Georgian Republic or Ghana or Gold Coast or Grenada or Guatemala or Guinea or Guiana or Guyana or Haiti or Honduras or India or Maldives or Indonesia or Iran or Iraq or Isle of Man or Jamaica or Jordan or Kazakhstan or Kazakh or Kenya or Kiribati or Korea or Kosovo or Kyrgyzstan or Kirghizia or Kyrgyz Republic or Kirghiz or Kirgizstan or "Lao PDR" or Laos or Lebanon or Lesotho or Basutoland or Liberia or Libya or Macedonia or Madagascar or Malagasy Republic or Malaysia or Malaya or Malay or Sabah or Sarawak or Malawi or Nyasaland or Mali or Marshall Islands or Mauritania or Mauritius or Agalega Islands or Mexico or Micronesia or Middle East or Moldova or Moldovia or Moldovian or Mongolia or Montenegro or Morocco or Ifni or Mozambique or Myanmar or Myanma or Burma or Namibia or Nepal or Nicaragua or Niger or Nigeria or Muscat or Pakistan or Palau or Palestine or Panama or Paraguay or Peru or Philippines or Philipines or Phillipines or Phillippines or Romania or Rumania or Roumania or Russia or Russian or Rwanda or Ruanda or Saint Lucia or St Lucia or Saint Vincent or St Vincent or Grenadines or Samoa or Samoan Islands or Navigator Island or Navigator Islands or Sao Tome or Senegal or Serbia or Montenegro or Seychelles or Sierra Leone or Sri Lanka or Ceylon or Solomon Islands or Somalia or Sudan or Suriname or Surinam or Swaziland or Syria or Tajikistan or Tadzhikistan or Tadjikistan or Tadzhik or Tanzania or Thailand or Togo or Togolese Republic or Tonga or Tunisia or Turkey or Turkmenistan or Turkmen or Uganda or Ukraine or USSR or Soviet Union or Union of Soviet Socialist Republics or Uzbekistan or Uzbek or Vanuatu or New Hebrides or Venezuela or Vietnam or Viet Nam or West Bank or Yemen or Yugoslavia or Zambia or Zimbabwe or Rhodesia).tw,kf

5. 1 AND 2 AND 3 AND 4

Table S1. Inclusion and exclusion criteria.

INCLUSION

- ☐ Low or middle income country setting
- ☐ Must include, but is not restricted to, an adolescent population (10-19 completed years)
- ☐ Reports on one of the following:
  - Types of food consumed
  - Food composition
  - Dietary diversity
  - Meal patterns
  - Place consumed (e.g. school, home, community center)
  - Frequency of consumption
  - Energy or macronutrient intake
  - Micronutrient intake
- ☐ Relevant study types/designs:
  - Observational studies (e.g. cross-sectional, cohort, case-control)
  - Randomized controlled trials (RCTs) with a control arm or baseline nutritional data
  - Surveys
  - Program evaluations
  - Qualitative research studies
  - Descriptive program/government documents
- ☐ Data collection in 2007 or later

EXCLUSION

- ☐ Population includes boys only; or the study does not disaggregate outcome data by sex
- ☐ Unhealthy study population (e.g. populations with chronic or genetic diseases such as HIV, TB, or metabolic disorders)
- ☐ Experimental study designs (e.g. RCTs) that do not have a "standard of care" or "usual practices" arm
- ☐ Non-English language

Table S2. Daily consumption (%) of each food group, by region.

	Middle East & North Africa	Latin America & Caribbean	Africa	Europe & Central Asia	South Asia	East Asia & Pacific	All
**Grains, white roots, tubers, plantains**							
N studies	2	1	3	NR	6	1	13
N participants	466	236	2694	NR	924	1186	5506
Daily consumption (%)	56%	82%	72%	NR	63%	100%	76%
**Pulses**							
N studies	NR	NR	3	NR	6	2	11
N participants	NR	NR	864	NR	849	1328	3041
Daily consumption (%)	NR	NR	36%	NR	84%	96%	76%
**Dairy**							
N studies	9	2	4	NR	8	3	26
N participants	4745	27,892	3055	NR	1483	1365	39,419
Daily consumption (%)	75%	0.4%	6%	NR	71%	88%	16%
**Meat, poultry, fish**							
N studies	3	NR	6	2	10	3	24
N participants	511	NR	3068	1206	1236	2054	8075
Daily consumption (%)	1%	NR	13%	63%	68%	83%	46%
**Fruits**							
N studies	9	5	4	NR	10	8	36
N participants	6508	19,759	804	NR	2376	7136	36,583
Daily consumption (%)	90%	9%	1%	NR	59%	99%	44%
**Vegetables**							
N studies	9	4	3	NR	10	8	34
N participants	4343	24,299	788	NR	2878	9622	41,930
Daily consumption (%)	82%	1%	5%	NR	72%	100%	37%
**Sweet snack foods**							
N studies	6	4	4	NR	3	1	18
N participants	4024	85,504	1303	NR	1064	335	92234
Daily consumption (%)	64%	NR	34%	NR	38%	100%	4%
**Salty snack foods**							
N studies	5	1	1	NR	1	1	9
N participants	2466	9222	3	NR	84	848	12,623
Daily consumption (%)	37%	100%	100%	NR	100%	100%	8%
**Fast foods**							
N studies	8	4	5	1	11	2	31
N participants	1880	1240	4223	459	4934	1452	13,106
Daily consumption (%)	8%	39%	21%	10%	25%	NR	20%
**Sugar-sweetened beverages**							
N studies	9	8	3	NR	6	4	30
N participants	2361	82,873	2705	NR	959	1112	90,010

Daily consumption (%)	33%	1%	7%	NR	94%	17%	4%

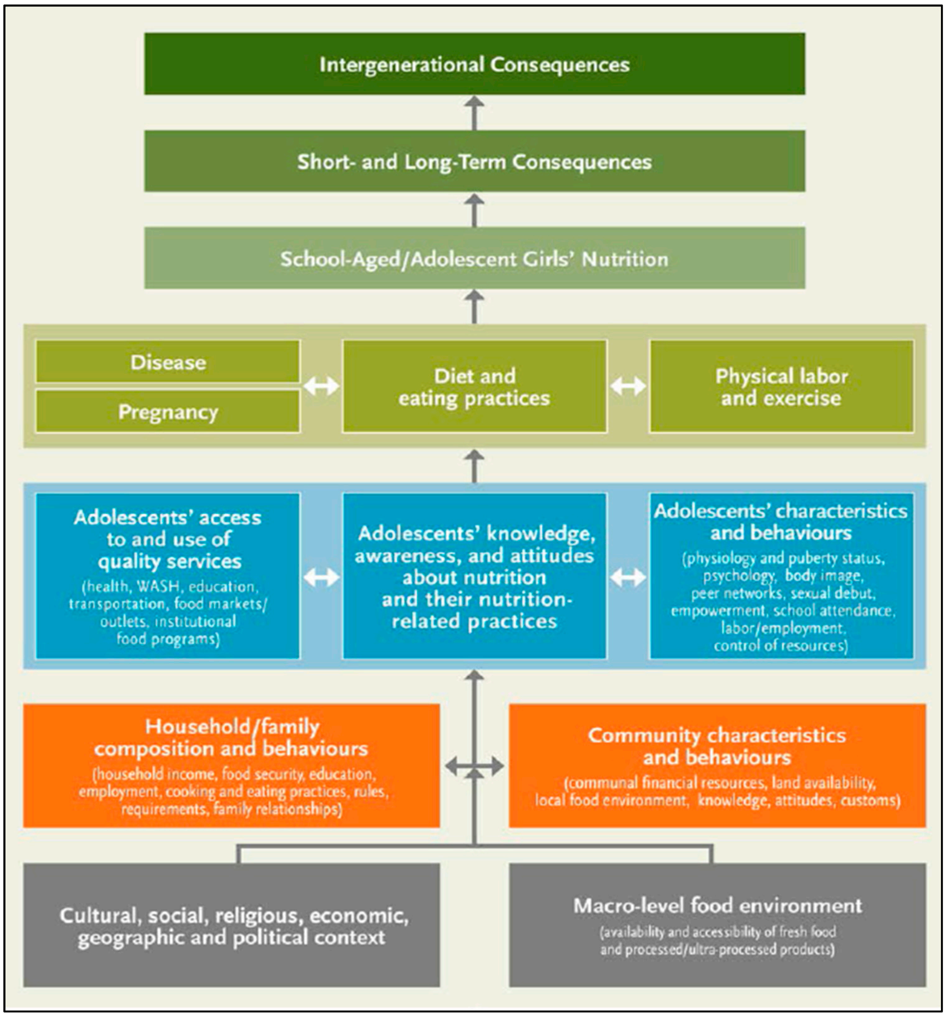

Figure S1. Conceptual framework outlining the determinants of adolescent girls' nutrition.
